# Association of Ratio of Apolipoprotein B to Apolipoprotein A1 With Survival in Peritoneal Dialysis

**DOI:** 10.3389/fnut.2022.801979

**Published:** 2022-03-25

**Authors:** Jing Yu, Xi Xia, Na-Ya Huang, Ya-Gui Qiu, Xiao Yang, Hai-Ping Mao, Wei Chen, Feng-Xian Huang

**Affiliations:** ^1^Department of Nephrology, The First Affiliated Hospital, Sun Yat-sen University, Guangzhou, China; ^2^Key Laboratory of Nephrology, National Health Commission and Guangdong Province, The First Affiliated Hospital, Sun Yat-sen University, Guangzhou, China

**Keywords:** apolipoprotein, peritoneal dialysis, survival, diabetes, atherosclerosis, cohort study

## Abstract

**Background:**

Although the ratio of apolipoprotein B (apo B) to apolipoprotein A1 (apo A1) (apo B/apo A1) seems to be associated with mortality in hemodialysis (HD) patients, the association of apo B/apo A1 ratio with death remains not clear in peritoneal dialysis (PD) patients.

**Aims:**

The study targets to examine the relationship of apo B/apo A1 ratio with survival in patients receiving PD treatment.

**Methods:**

In this single-center prospective observational cohort study, we enrolled 1,616 patients receiving PD treatment with a median follow-up time of 47.6 months. We used a multivariable Cox proportional hazards model to examine the relationship between apo B/apo A1 ratio and cardiovascular (CV) and all-cause mortality. The association of apo B/apo A1 ratio with atherosclerotic and non-atherosclerotic CV mortality was further evaluated by competing risk regression models.

**Results:**

During the follow-up, 508 (31.4%) patients died, 249 (49.0%) died from CV events, of which 149 (59.8%) were atherosclerotic CV mortality. In multivariable models, for 1-SD increase in apo B/apo A1 ratio level, the adjusted hazard ratios for CV and all-cause mortality were 1.26 [95% confidence interval (CI), 1.07–1.47; *P* = 0.005] and 1.20 (95% CI, 1.07–1.35; *P* = 0.003), respectively. The adjusted subdistribution hazard ratios for atherosclerotic and non-atherosclerotic CV mortality were 1.43 (95% CI, 1.19–1.73; *P* < 0.001) and 0.85 (95% CI, 0.64–1.13; *P* = 0.256), respectively. For quartile analysis, patients in quartile 4 had higher CV, all-cause, and atherosclerotic CV mortality compared with those in quartile 1. Moreover, apo B/apo A1 ratio had a diabetes-related difference in CV, all-cause, and atherosclerotic CV mortality.

**Conclusion:**

Elevated apo B/apo A1 ratio level was significantly associated with CV, all-cause, and atherosclerotic CV mortality in patients undergoing PD. Moreover, the association was especially statistically significant in patients with diabetes.

## Introduction

Cardiovascular (CV) events have so far been the biggest obstacle to the survival of patients with end-stage renal disease (ESRD) (1). Even if some known CV risk factors have been controlled, including blood pressure, glucose and low-density lipoprotein cholesterol (LDL-C), the risk of mortality in patients with ESRD still remains at a high level (2, 3). Dyslipidemia, rather than hyperlipidemia, may play an important role in atherosclerotic CV events and mortality (4). Due to the inhibition of lipoprotein lipase and hepatic lipase activity in the extrinsic and intrinsic lipid metabolism pathways by uremic toxins, patients with ESRD showed a decrease in high-density lipoprotein cholesterol (HDL-C) levels and an increase in triglycerides (TG) levels on the characteristics of the lipid profile (4).

A genetic study by Sarwar et al. and a meta-analysis involving fibrates supported the causal relationship between hypertriglyceridemia and atherosclerotic CV events (5, 6). However, it is TG-rich lipoproteins instead of TG *per se* that determines atherosclerosis in hypertriglyceridemia (7). Apolipoprotein B (apo B) is an important structural protein of all lipoprotein particles with atherogenic effect, and each particle carries only one apo B molecule (8). This feature enables apo B to reliably reflect the atherogenic effect in plasma than LDL-C or TG-rich lipoprotein cholesterol alone. HDL-C is generally recognized to be with anti-atherosclerotic effect and is beneficial to CV health (9). Apolipoprotein A1 (apo A1), which accounts for 60%–70% of all apolipoproteins carried by HDL-C, is also considered to be with anti-atherosclerotic effect (10). Therefore, apo B/apo A1 ratio reflects the balance between pro-atherosclerotic and anti-atherosclerotic effect in plasma.

The relationship between some conventional lipid parameters that can reflect this balance effect and prognosis of ESRD patients remains controversial. A study conducted by Chang et al. concluded that elevated TG/HDL-C ratio was associated with better CV and overall survival in patients on hemodialysis (HD) (11). A study from our center showed that a higher TG/HDL-C ratio was associated with an increased risk of CV and all-cause mortality in peritoneal dialysis (PD) patients (12). Studies from Little et al. and Noh et al. found that increased total cholesterol (TC)/HDL-C ratio was associated with mortality in PD patients, but Noh et al. did not observe an association between TC/HDL-C ratio and CV mortality (13, 14). However, studies focused on the relationship between apo B/apo A1 ratio and survival in chronic kidney disease (CKD) or ESRD patients are rare. Two studies based on prevalent HD patients conducted by Sato et al. and Kaysen et al. came to completely different conclusions (15, 16). In terms of PD, although only two small-sample retrospective cohort studies have reached conclusions consistent with the study conducted by Sato et al. (17, 18), they have not further explored whether different causes of CV mortality have an effect on the relationship of apo B/apo A1 ratio with CV mortality. The lipid profile of PD has been reported to be more atherogenic than that of HD inherently (19). Therefore, we assumed that higher apo B/apo A1 ratio level was not only an independent risk factor of CV and all-cause mortality, but also independently associated with atherosclerotic CV mortality. The study targeted to evaluate the relationship between apo B/apo A1 ratio and survival in a large sample of PD population.

## Materials and Methods

### Study Design and Population

A prospective observational cohort study of 1,882 patients diagnosed with ESRD who began PD therapy between January 1, 2006 and December 31, 2013 in the First Affiliated Hospital of Sun Yat-sen University (SYSU) was performed. Individuals who met the inclusion criteria included those who were older than 18 years old, maintained PD therapy for at least 3 months, and signed a written informed consent. Patients who met the exclusion criteria included those who had maintained HD therapy for at least 3 months, who had suffered from malignant diseases, those with a history of failed kidney transplantation, and those with incomplete lipid data. In the end, only 1,616 patients entered the study, and the deadline for the follow-up was June 30, 2019. The entire study was carried out in line with the ethical principles of the Declaration of Helsinki and was approved by the Clinical Research Ethics Committee of the First Affiliated Hospital of SYSU. Written informed consent were obtained before all participants entered the study.

### Data Collection and Measurements

When the patient was admitted to hospital, data on epidemiology and concomitant diseases would be collected. Individuals who met the diagnostic criteria for diabetes or using oral hypoglycemic drugs or insulin were believed to have diabetes. Patients who suffered from at least one of the following events were believed to have a history of CV events: angina, myocardial infarction, congestive heart failure, percutaneous coronary intervention, coronary artery bypass, or ischemic/hemorrhagic stroke (20). Individuals whose blood pressure measurements exceeded 140/90 mmHg at least twice in different time periods in a quiet state or took antihypertensive drugs were considered to have hypertension.

Laboratory data were collected within 3 months of the start of PD treatment. Lipid parameters including TC, TG, LDL-C, HDL-C, apo A1, and apo B levels, as well as other biochemical indexes, were measured in the First Affiliated Hospital of SYSU (21). We adopted the immunoturbidimetric method to measure the levels of apo B and apo A1, and standardized methods were applied to measure other laboratory variables. Clinical data (e.g., body mass index (BMI), blood pressure, and medication) were collected simultaneously. Estimated glomerular filtration rate (eGFR), served as an indicator to assess residual renal function, was calculated by CKD-EPI formula. Kt/V is an important parameter for assessing the adequacy of dialysis, which the value can be calculated by PD Adequest software (Baxter Healthcare, Guangzhou, China). We consider that the dialysis adequacy is not up to standard if Kt/V level is less than 1.7. In the PD process, we would select peritoneal dialysate with appropriate glucose concentration (1.5%, 2.5%, or 4.25%, Baxter Healthcare, Guangzhou, China) for dialysis according to the specific situation of the patients.

### Follow-Up

The primary and the secondary endpoint of this study was CV and all-cause mortality, respectively. Atherosclerotic and non-atherosclerotic mortality was further differentiated on the basis of the etiology of CV mortality. The definitions and the judgment methods of the endpoints have been described in our previous study exhaustively (21). Follow-up team composed of physicians and well-trained nurses conducted a telephone or face-to-face interview with the patients monthly to evaluate the general conditions and accordingly adjust medication prescription. Meanwhile, patients needed to return to the center every 3 months to receive a comprehensive medical evaluation as requested (21).

### Statistical Analysis

The participants were categorized into quartiles according to apo B/apo A1 ratio levels: quartile 1, < 0.52; quartile 2, 0.52–0.66; quartile 3, 0.66–0.84; and quartile 4, > 0.84. Apo B, apo A1, and some conventional lipid parameters were also categorized into quartiles according to their baseline levels. Means ± standard deviations (SDs) are expressed for normally distributed variables, medians and 25th–75th percentiles are expressed for non-parametric variables. Frequencies and percentages are expressed for categorical parameters.

Kaplan–Meier survival curves were performed to analyze CV and overall survival, and the method to assess the distributions of survival among apo B/apo A1 ratio quartiles was a log-rank test. We explored the shape of the relationship between apo B/apo A1 ratio and CV and all-cause mortality by using a restricted cubic spline model with four knots. A Cox proportional hazards model was used to analyze the independent association of apo B/apo A1 ratio with CV and all-cause mortality. The proportional hazards assumptions were tested by Schoenfeld residual plots. Hazard ratio (HR) with 95% confidence interval (CI) was used to present the results. Since atherosclerotic and non-atherosclerotic CV mortality are competing events, cumulative incidence function (CIF) curves were performed to analyze cumulative incidence of these two outcomes, and the method to assess the distributions of incidence among apo B/apo A1 ratio quartiles was a Fine-Gray test. The association between apo B/apo A1 ratio and atherosclerotic and non-atherosclerotic CV mortality was evaluated by competing risk regression models. Subdistribution hazard ratio (SHR) with 95% CI was used to present the results.

Subgroup analyses were conducted to further examine the relationship between apo B/apo A1 ratio and mortality based on several groups of clinical parameters by forest plots. A formal interaction test was carried out to investigate the interaction between diabetes and apo B/apo A1 ratio. The standard of statistical significance is *P* < 0.05. All analyses were carried through SPSS software, version 13.0 (IBM Corp., Chicago, IL, United States) and Stata software, version 14.0 (Stata Corp., College Station, TX, United States).

## Results

### Participants

**FIGURE 1:**
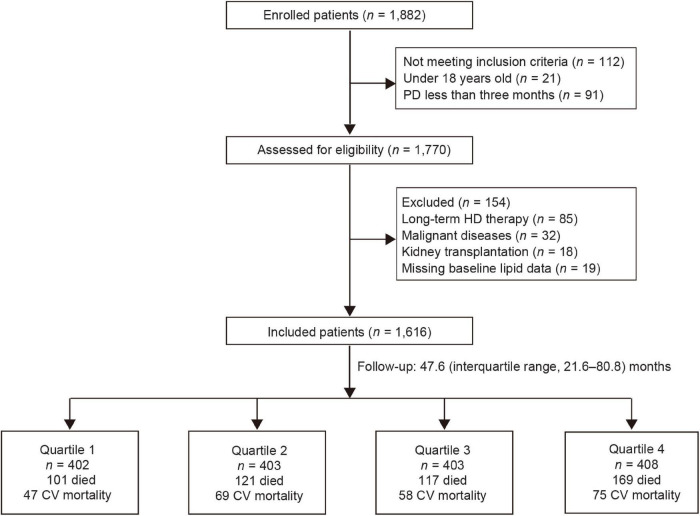
Flow chart for the study. CV, cardiovascular; HD, hemodialysis; PD, peritoneal dialysis.

Among 1,882 patients treated with PD, 1,616 eligible patients entered the final study (Figure 1). Baseline demographic and clinical and laboratory data of the overall population, as well as patients divided by quartiles of apo B/apo A1 ratio, are summarized in Table 1 (mean age, 47.5 ± 15.2 years, men accounted for 59.8%, and 25.7% had a history of diabetes). The median of apo B/apo A1 ratio was 0.66 (interquartile range, 0.52–0.84). Patients in the highest quartile (quartile 4) tended to be older, suffered more from diabetes, were more likely to have higher BMI, TC, TG, LDL-C, and hypersensitive C-reactive protein (hs-CRP) levels but lower serum albumin and HDL-C levels.

**TABLE 1:** Baseline characteristics of the study patients according to apo B/apo A1 ratio quartiles.

Variables	Total (*n* = 1,616)	Apo B/apo A1 ratio quartiles			
		Q1 (<0.52) (*n* = 402)	Q2 (0.52–0.66) (*n* = 403)	Q3 (0.66–0.84) (*n* = 403)	Q4 (>0.84) (*n* = 408)
Age (y)	47.5 ± 15.2	44.8 ± 15.0	46.6 ± 15.2	47.8 ± 14.5	50.7 ± 15.6
Male *n* (%)	966 (59.8)	220 (54.7)	259 (64.3)	244 (60.5)	243 (59.6)
Diabetes *n* (%)	415 (25.7)	77 (19.2)	98 (24.3)	106 (26.3)	134 (32.8)
History of CV events *n* (%)	596 (36.9)	143 (35.6)	135 (33.5)	154 (38.2)	164 (40.2)
Hypertension *n* (%)	1,439 (89.0)	362 (90.0)	358 (88.8)	365 (90.6)	354 (86.8)
BMI (kg/m^2^)	21.6 ± 3.1	20.5 ± 2.6	21.4 ± 3.1	22.1 ± 3.1	22.4 ± 3.3
SBP (mmHg)	136.5 ± 19.8	136.3 ± 20.1	136.7 ± 19.8	135.7 ± 18.5	137.3 ± 20.6
DBP (mmHg)	84.7 ± 14.4	86.1 ± 15.6	84.4 ± 14.1	84.8 ± 13.3	83.5 ± 14.5
Hemoglobin (g/L)	105.0 ± 21.2	103.5 ± 22.0	105.3 ± 20.9	107.3 ± 21.0	103.8 ± 20.8
Serum albumin (g/L)	37.3 ± 5.2	37.3 ± 5.0	37.2 ± 5.0	37.9 ± 5.0	36.8 ± 5.6
Hs-CRP (mg/L)	1.75 [0.64–5.74]	1.02 [0.40–3.01]	1.59 [0.59–4.26]	1.87 [0.69–7.03]	3.58 [1.11–10.04]
eGFR (mL/min/1.73 m^2^)	6.8 ± 3.1	6.8 ± 2.9	7.1 ± 3.5	6.7 ± 3.2	6.8 ± 2.9
Total Kt/V	2.5 ± 0.7	2.4 ± 0.6	2.5 ± 0.8	2.5 ± 0.7	2.5 ± 0.7
TC (mg/dL)	196.4 ± 51.2	180.8 ± 41.7	188.2 ± 43.1	197.3 ± 47.5	218.9 ± 61.6
TG (mg/dL)	124.8 [89.4–177.0]	100.0 [71.7–137.2]	116.8 [85.8–163.7]	128.3 [94.7–180.5]	164.2 [116.2–229.2]
HDL-C (mg/dL)	47.6 ± 14.9	57.5 ± 15.2	49.3 ± 13.0	44.7 ± 13.4	39.2 ± 11.6
LDL-C (mg/dL)	113.4 ± 38.7	97.8 ± 30.1	106.5 ± 31.4	116.7 ± 35.1	132.1 ± 47.1
Apo B (mg/dL)	85.0 [71.0–101.8]	69.0 [58.0–81.0]	80.0 [70.0–95.0]	89.0 [78.0–102.0]	106.5 [91.0–124.8]
Apo A1 (mg/dL)	127.0 [105.0–156.0]	163.0 [137.8–193.3]	137.0 [117.0–157.0]	119.0 [105.0–140.0]	103.0 [87.0–118.0]
Apo B/apo A1 ratio	0.66 [0.52–0.84]	0.44 [0.39–0.48]	0.59 [0.55–0.63]	0.74 [0.70–0.78]	0.99 [0.91–1.17]
Statin use *n* (%)	236 (14.6)	48 (11.9)	57 (14.1)	74 (18.4)	57 (14.0)

*Means ± standard deviations (SDs) are expressed for normally distributed variables, medians and 25th–75th percentiles are expressed for non-parametric variables. Frequencies and percentages are expressed for categorical parameters. Kt/V is an important parameter for assessing the adequacy of dialysis, if the value is less than 1.7, the dialysis adequacy is thought to be not up to standard. Conversion factors for units: TC, HDL-C, and LDL-C in mg/dL to mmol/L, × 0.0259; TG in mg/dL to mmol/L, × 0.0113; Apo B and apo A1 in mg/dL to g/L, × 0.01.*

*Apo A1, apolipoprotein A1; Apo B, apolipoprotein B; BMI, body mass index; CV, cardiovascular; DBP, diastolic blood pressure; eGFR, estimated glomerular filtration rate; HDL-C, high-density lipoprotein cholesterol; Hs-CRP, hypersensitive C-reactive protein; LDL-C, low-density lipoprotein cholesterol; Q1–Q4, lowest to highest quartile; SBP, systolic blood pressure; TC, total cholesterol; TG, triglycerides.*

The median follow-up time was 47.6 (interquartile range, 21.6–80.8) months, and 508 (31.4%) patients died. The leading cause of death was CV mortality (249, 49.0%). The proportion of atherosclerotic and non-atherosclerotic CV mortality was 59.8% vs. 40.2%, respectively. Other causes of death were listed in Supplementary Table 1. In addition to death, other clinical outcomes of these patients consisted of continued PD treatment (280, 17.3%), kidney transplant (389, 24.1%), change the dialysis method to HD (299, 18.5%), went to other centers for treatment (75, 4.6%), and loss to follow-up (65, 4.0%).

### Apolipoprotein B/Apolipoprotein A1 Ratio and Cardiovascular and All-Cause Mortality

**FIGURE 2:**
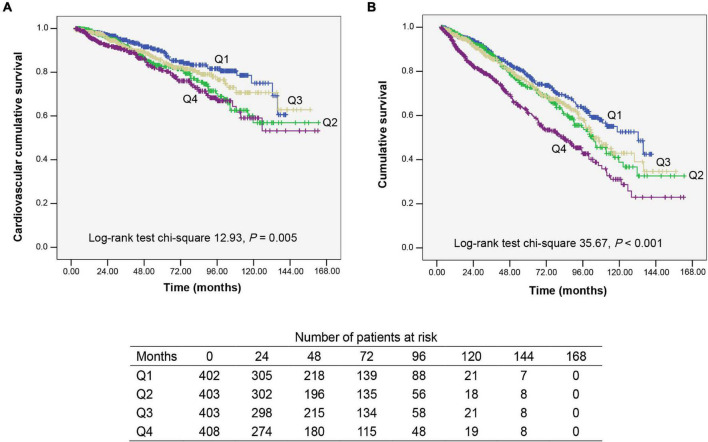
Kaplan–Meier survival curves for participants categorized by apolipoprotein B (apo B)/apolipoprotein A1 (apo A1) ratio quartiles. **(A)** Cardiovascular mortality survival curves divided by apo B/apo A1 ratio quartiles. **(B)** All-cause mortality survival curves divided by apo B/apo A1 ratio quartiles. Q1–Q4, lowest to highest quartile.

Kaplan–Meier analysis is used to assess CV and overall survival among quartiles of apo B/apo A1 ratio levels (Figure 2). Patients in quartile 4 had the lowest CV and overall survival rate when compared with the other three lower quartiles (*P* = 0.005 and *P* < 0.001, respectively).

**FIGURE 3:**
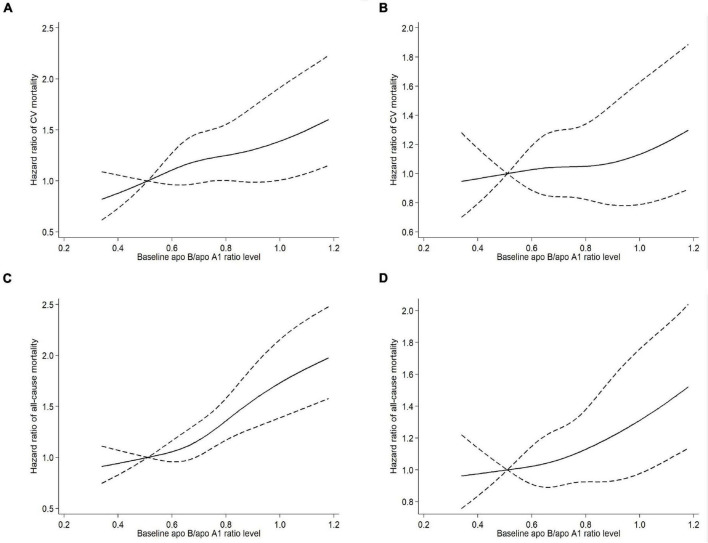
Univariable **(A)** and multivariable **(B)** adjusted hazard ratios of CV mortality, univariable **(C)** and multivariable **(D)** adjusted hazard ratios of all-cause mortality associated with apo B/apo A1 ratio levels in Cox model using restricted cubic splines, adjusted for age, sex, diabetes, a history of CV events, body mass index, systolic blood pressure, hemoglobin, serum albumin, hypersensitive C-reactive protein, estimated glomerular filtration rate, total Kt/V, and statin use. Apo A1, apolipoprotein A1; Apo B, apolipoprotein B; CV, cardiovascular.

A trend toward linear relationship between apo B/apo A1 ratio and CV and all-cause mortality was observed when we examined apo B/apo A1 ratio as a continuous variable in restricted cubic spline models (Figure 3). A multivariable Cox regression analysis was performed to determine the association of apo B/apo A1 ratio with CV and all-cause mortality, as listed in Table 2. In the final adjusted model, the adjusted HRs for 1-SD increase in apo B/apo A1 ratio level for CV and all-cause mortality were 1.26 (95% CI, 1.07–1.47; *P* = 0.005) and 1.20 (95% CI, 1.07–1.35; *P* = 0.003), respectively. For quartile analysis, a statistically significant association existed between quartile 4 and CV (HR, 1.79; 95% CI, 1.12–2.84; *P* = 0.015) and all-cause (HR, 1.57; 95% CI, 1.15–2.15; *P* = 0.005) mortality relative to quartile 1.

**TABLE 2:** Association of apo B/apo A1 ratio with CV and all-cause mortality.

	HR (95% CI) per 1-SD increase		Q2 (*n* = 403)		Q3 (*n* = 403)		Q4 (*n* = 408)	
		*P*-value	HR (95% CI)	*P*-value	HR (95% CI)	*P*-value	HR (95% CI)	*P*-value
**CV mortality**								
Unadjusted	1.26 (1.14–1.38)	<0.001	1.59 (1.10–2.30)	0.015	1.30 (0.89–1.91)	0.180	1.88 (1.30–2.70)	0.001
Model 1^a^	1.14 (1.04–1.26)	0.006	1.45 (1.00–2.10)	0.051	1.15 (0.78–1.69)	0.487	1.50 (1.04–2.17)	0.029
Model 2^b^	1.13 (1.02–1.26)	0.026	1.34 (0.92–1.95)	0.132	1.02 (0.69–1.51)	0.921	1.41 (0.97–2.05)	0.073
Model 3^c^	1.23 (1.05–1.44)	0.011	1.71 (1.10–2.65)	0.017	1.16 (0.73–1.84)	0.539	1.68 (1.07–2.66)	0.025
Model 4^d^	1.26 (1.07–1.47)	0.005	1.74 (1.12–2.70)	0.014	1.19 (0.75–1.90)	0.461	1.79 (1.12–2.84)	0.015
**All-cause mortality**								
Unadjusted	1.27 (1.19–1.36)	<0.001	1.31 (1.00–1.70)	0.049	1.23 (0.95–1.61)	0.123	1.99 (1.56–2.55)	<0.001
Model 1^a^	1.16 (1.09–1.24)	<0.001	1.22 (0.93–1.58)	0.150	1.09 (0.84–1.43)	0.515	1.59 (1.24–2.04)	<0.001
Model 2^b^	1.15 (1.07–1.24)	<0.001	1.15 (0.88–1.50)	0.309	1.01 (0.77–1.33)	0.925	1.53 (1.19–1.97)	0.001
Model 3^c^	1.19 (1.06–1.34)	0.004	1.21 (0.89–1.65)	0.227	0.98 (0.71–1.34)	0.895	1.54 (1.13–2.10)	0.006
Model 4^d^	1.20 (1.07–1.35)	0.003	1.22 (0.89–1.66)	0.212	0.99 (0.72–1.36)	0.946	1.57 (1.15–2.15)	0.005

*We indicated the lowest quartile (Q1) as the reference group (n = 402).*

*Apo A1, apolipoprotein A1; Apo B, apolipoprotein B; CI, confidence interval; CV, cardiovascular; HR, hazard ratio; Q1–Q4, lowest to highest quartile; SD, standard deviation.*

*^a^Age and sex were put into the model.*

*^b^Age, sex, diabetes, a history of cardiovascular events, body mass index, and systolic blood pressure were put into the model.*

*^c^Age, sex, diabetes, a history of cardiovascular events, body mass index, systolic blood pressure, hemoglobin, serum albumin, hypersensitive C-reactive protein, estimated glomerular filtration rate, and total Kt/V were put into the model.*

*^d^Age, sex, diabetes, a history of cardiovascular events, body mass index, systolic blood pressure, hemoglobin, serum albumin, hypersensitive C-reactive protein, estimated glomerular filtration rate, total Kt/V, and statin use were put into the model.*

With regard to apo B or apo A1 alone or some conventional lipid parameters, apo B was significantly associated with CV and all-cause mortality for continuous variable analysis, however, the quartile analysis did not show statistical significance for all-cause mortality. No association was found between apo A1 and worse prognosis. The association of LDL-C/HDL-C ratio with mortality exhibited similar pattern to apo B/apo A1 ratio. TC/HDL-C ratio level was significantly associated with CV and all-cause mortality for continuous variable analysis, but the quartile analysis did not show statistical significance for CV mortality (Supplementary Table 2).

### Apolipoprotein B/Apolipoprotein A1 Ratio and Atherosclerotic and Non-atherosclerotic Cardiovascular Mortality

**FIGURE 4:**
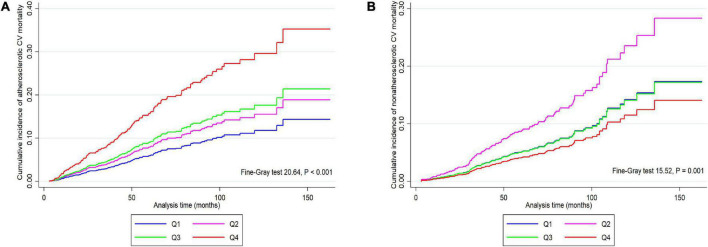
Cumulative incidence function curves for the cumulative incidence of atherosclerotic **(A)** and non-atherosclerotic **(B)** CV mortality in participants categorized by apolipoprotein B/apolipoprotein A1 ratio quartiles. CV, cardiovascular; Q1–Q4, lowest to highest quartile.

The CIF curves were shown in Figure 4, we found that patients in quartile 4 had higher cumulative incidence risk of atherosclerotic CV mortality (*P* < 0.001) but lower incidence risk of non-atherosclerotic CV mortality (*P* = 0.001) than those in other three lower quartiles.

The relationship of apo B/apo A1 ratio with atherosclerotic and non-atherosclerotic CV mortality was further investigated by competing risk regression models in order to clarify the impact of different etiologies on the association of apo B/apo A1 ratio with CV mortality. As shown in Table 3, in the fully adjusted model, the adjusted SHRs for 1-SD increase in apo B/apo A1 ratio level for atherosclerotic and non-atherosclerotic CV mortality were 1.43 (95% CI, 1.19–1.73; *P* < 0.001) and 0.85 (95% CI, 0.64–1.13; *P* = 0.256), respectively. For quartile analysis, apo B/apo A1 ratio in quartile 4 was significantly associated with a higher risk of atherosclerotic CV (SHR, 2.21; 95% CI, 1.16–4.23; *P* = 0.016) but not non-atherosclerotic CV (SHR, 0.99; 95% CI, 0.41–2.37; *P* = 0.979) mortality relative to quartile 1.

**TABLE 3:** Association of apo B/apo A1 ratio with atherosclerotic and non-atherosclerotic CV mortality.

	SHR (95% CI) per 1-SD increase		Q2 (*n* = 403)		Q3 (*n* = 403)		Q4 (*n* = 408)	
		*P*-value	SHR (95% CI)	*P*-value	SHR (95% CI)	*P*-value	SHR (95% CI)	*P*-value
**Atherosclerotic CV mortality**								
Unadjusted	1.41 (1.27–1.57)	<0.001	1.35 (0.79–2.29)	0.271	1.55 (0.93–2.60)	0.094	2.81 (1.75–4.51)	<0.001
Model 1^a^	1.29 (1.14–1.46)	<0.001	1.21 (0.71–2.09)	0.484	1.36 (0.80–2.31)	0.253	2.32 (1.43–3.77)	0.001
Model 2^b^	1.34 (1.21–1.48)	<0.001	1.16 (0.66–2.02)	0.604	1.21 (0.70–2.09)	0.487	2.27 (1.37–3.75)	0.001
Model 3^c^	1.39 (1.16–1.68)	<0.001	1.41 (0.75–2.64)	0.290	1.21 (0.64–2.29)	0.548	1.98 (1.09–3.62)	0.026
Model 4^d^	1.43 (1.19–1.73)	<0.001	1.46 (0.76–2.81)	0.253	1.30 (0.67–2.49)	0.437	2.21 (1.16–4.23)	0.016
**Non-atherosclerotic CV mortality**								
Unadjusted	0.82 (0.66–1.01)	0.064	1.75 (1.04–2.95)	0.034	0.99 (0.55–1.78)	0.972	0.80 (0.43–1.49)	0.479
Model 1^a^	0.74 (0.59–0.93)	0.010	1.57 (0.93–2.66)	0.093	0.87 (0.49–1.57)	0.651	0.63 (0.32–1.20)	0.160
Model 2^b^	0.70 (0.55–0.90)	0.006	1.47 (0.86–2.50)	0.154	0.81 (0.45–1.47)	0.495	0.58 (0.29–1.14)	0.113
Model 3^c^	0.86 (0.64–1.14)	0.283	2.19 (1.08–4.46)	0.031	1.17 (0.53–2.55)	0.702	1.01 (0.42–2.44)	0.974
Model 4^d^	0.85 (0.64–1.13)	0.256	2.21 (1.08–4.51)	0.029	1.16 (0.53–2.54)	0.715	0.99 (0.41–2.37)	0.979

*We indicated the lowest quartile (Q1) as the reference group (n = 402).*

*Apo A1, apolipoprotein A1; Apo B, apolipoprotein B; CI, confidence interval; CV, cardiovascular; Q1–Q4, lowest to highest quartile; SD, standard deviation; SHR, subdistribution hazard ratio.*

*^a^Age and sex were put into the model.*

*^b^Age, sex, diabetes, a history of cardiovascular events, body mass index, and systolic blood pressure were put into the model.*

*^c^Age, sex, diabetes, a history of cardiovascular events, body mass index, systolic blood pressure, hemoglobin, serum albumin, hypersensitive C-reactive protein, estimated glomerular filtration rate, and total Kt/V were put into the model.*

*^d^Age, sex, diabetes, a history of cardiovascular events, body mass index, systolic blood pressure, hemoglobin, serum albumin, hypersensitive C-reactive protein, estimated glomerular filtration rate, total Kt/V, and statin use were put into the model.*

With regard to apo B or apo A1 alone or some conventional lipid parameters, increased apo B, LDL-C/HDL-C ratio, and TC/HDL-C ratio levels were significantly associated with an increased risk of atherosclerotic CV mortality, but had no association with non-atherosclerotic CV mortality. Apo A1 levels were not associated with both the two different etiologies of CV mortality (Supplementary Table 3).

### Interaction Between Diabetes and Apolipoprotein B/Apolipoprotein A1 Ratio on Mortality

Next, subgroup analyses were carried out and we found that the CV, all-cause, and atherosclerotic CV mortality significantly increased with the elevation of apo B/apo A1 ratio levels in all predesignated subgroups except in patients without diabetes (Supplementary Figure 1).

We then proceeded a formal interaction test to examine whether the association of apo B/apo A1 ratio with survival differed due to diabetes. The results indicated a significant interaction between diabetes and apo B/apo A1 ratio on CV (*P* = 0.035), all-cause (*P* = 0.012), and atherosclerotic CV (*P* = 0.011) mortality, but not on non-atherosclerotic CV (*P* = 0.498) mortality (data not shown). As shown in Table 4, with each 1-SD increase in apo B/apo A1 ratio level in patients with diabetes, the adjusted HRs for CV and all-cause mortality were 1.61 (95% CI, 1.27–2.05; *P* < 0.001) and 1.49 (95% CI, 1.25–1.78; *P* < 0.001), respectively, the adjusted SHRs for atherosclerotic and non-atherosclerotic CV mortality were 1.96 (95% CI, 1.43–2.68; *P* < 0.001) and 0.79 (95% CI, 0.57–1.12; *P* = 0.184), respectively. However, we did not observe an association between apo B/apo A1 ratio and mortality in patients without diabetes.

**TABLE 4:** Mortality outcome for each 1-SD increase in apo B/apo A1 ratio level by diabetes.

Mortality outcome	Diabetics		Non-diabetics	
	HR (95% CI)	*P*-value	HR (95% CI)	*P-*value
**CV mortality**				
Unadjusted	1.23 (1.09–1.38)	0.001	1.11 (0.93–1.34)	0.258
Final model^a^	1.61 (1.27–2.05)	<0.001	1.06 (0.84–1.34)	0.636
**All-cause mortality**				
Unadjusted	1.22 (1.12–1.33)	<0.001	1.20 (1.07–1.35)	0.002
Final model^a^	1.49 (1.25–1.78)	<0.001	1.03 (0.87–1.22)	0.742
	**SHR (95% CI)**	***P*-value**	**SHR (95% CI)**	***P-*value**
**Atherosclerotic CV mortality**				
Unadjusted	1.36 (1.21–1.54)	<0.001	1.31 (1.09–1.58)	0.004
Final model^a^	1.96 (1.43–2.68)	<0.001	1.06 (0.80–1.40)	0.690
**Non-atherosclerotic CV mortality**				
Unadjusted	0.72 (0.57–0.92)	0.008	0.82 (0.58–1.18)	0.287
Final model^a^	0.79 (0.57–1.12)	0.184	0.97 (0.64–1.46)	0.877

*Apo A1, apolipoprotein A1; Apo B, apolipoprotein B; CI, confidence interval; CV, cardiovascular; HR, hazard ratio; SD, standard deviation; SHR, subdistribution hazard ratio.*

*^a^Adjusted for age, sex, a history of cardiovascular events, body mass index, systolic blood pressure, hemoglobin, serum albumin, hypersensitive C-reactive protein, estimated glomerular filtration rate, total Kt/V, and statin use.*

## Discussion

The results of this study indicated that elevated apo B/apo A1 ratio was significantly associated with CV, all-cause, and atherosclerotic CV mortality among patients receiving PD treatment. Additionally, subgroup analyses and formal interaction tests confirmed that the higher CV (HR, 1.61; 95% CI, 1.27–2.05; *P* < 0.001), all-cause (HR, 1.49; 95% CI, 1.25–1.78; *P* < 0.001), and atherosclerotic CV (SHR, 1.96; 95% CI, 1.43–2.68; *P* < 0.001) mortality was closely associated with the elevation of apo B/apo A1 ratio levels in patients with diabetes.

Kaysen et al. recruited 433 prevalent patients treated with HD and concluded that apo B/apo A1 ratio had no association with CV mortality (15), while a study from Japan including 1,081 prevalent patients undergoing HD demonstrated that apo B/apo A1 ratio was an independent risk factor of CV (HR, 1.38; 95% CI, 1.11–1.71; *P* = 0.004) and all-cause (HR, 1.16; 95% CI, 1.00–1.35; *P* = 0.046) mortality (16). Two small-sample retrospectively cohort studies conducted on PD patients also supported the positive association of apo B/apo A1 ratio with CV mortality (17, 18), which was consistent with our conclusions. Heterogeneity exists between PD and HD. For PD patients, most of the substrate required for increased lipoprotein synthesis are derived from a large amount of glucose absorbed by long-term exposure to dextrose-containing peritoneal dialysate (22). On the other hand, continuous loss of protein in peritoneal dialysate increases the compensatory synthesis of protein by liver similar to that of nephrotic syndrome (23, 24). These factors may make the lipid profile of PD patients more atherogenic than HD patients, which was supported by the results of higher apo B/apo A1 ratio, apo B, LDL-C and TG levels, and lower apo A1 and HDL-C levels in our study compared with the cohort in Sato et al. study (16). The study of Little et al. showed that the atherogenic condition of the lipid profile of PD patients worsened over time (13), which may be associated with the different etiology spectrum of CV mortality between PD and HD patients. Different etiology spectrum may contribute to the relationship between lipid parameters and prognosis in patients with ESRD. Our study pointed out that atherosclerotic events accounted for the majority of CV mortality in patients undergoing PD, the pathophysiological basis of which is mainly atherosclerosis and vascular occlusion caused by the formation and rupture of fibrous plaque (25). Increased lipoproteins enter the intima and promote the progression of atherosclerosis by depositing their cholesterol components in the atherosclerotic lesions to form fibrous plaques (26). When the plaque ruptures, it may occur vascular occlusive atherosclerotic events. In terms of HD, it has been reported that the main causes of CV mortality are non-atherosclerotic CV events (1, 27), the pathophysiological basis of which is mainly structural and functional cardiac abnormalities caused by increased arteriosclerosis (25). Anemia, calcium and phosphorus metabolism disorders, chronic inflammation, and increased advanced glycation end products, which are common to patients with ESRD, were closely associated with increased thickness and stiffness of arterial wall (25, 28, 29). Arteriosclerosis eventually leads to diastolic and systolic dysfunction and congestive heart failure (25). Two studies from the Study of Heart and Renal Protection (SHARP) trial, demonstrated that those lipoprotein components with pro-atherosclerotic effect indeed had a positive association with atherosclerotic CV mortality but negatively related to non-atherosclerotic CV mortality (30, 31). The competing risk regression analysis in our study showed that apo B/apo A1 ratio was positively associated with atherosclerotic CV mortality but was not associated with non-atherosclerotic CV mortality. All of these findings indicated that it is very necessary to distinct the etiology of CV mortality when exploring the relationship between lipid parameters and CV mortality in ESRD patients.

In addition to etiology, follow-up time is another important factor (32). Our study and Chmielewski et al. observed that higher apo B/apo A1 ratio level was associated with a survival advantage during 1 year follow-up period but poor clinical outcomes during nearly 4 years of follow-up (33). We speculated that apo B/apo A1 ratio was more likely to reflect energy metabolism and nutritional status in the short-term (34), but the pro-atherosclerotic effect gradually took effect over time. It is warranted to monitor the dynamic changes of the apo B/apo A1 ratio during the follow-up periods and carry out a time-stratified Cox regression analysis in the future.

Several studies have mentioned that apo B/apo A1 ratio was closely related to diabetes (35, 36), but whether the ratio had a diabetes-related difference in mortality is still unknown. As far as we know, our study is the first to examine the association of apo B/apo A1 ratio with survival by diabetes in patients with ESRD. Insulin resistance (IR) is a key feature common to type 2 diabetes (37). It can reduce insulin-mediated inhibition of hormone-sensitive lipase activity, which leads to lipolysis in adipose tissue, which increases free fatty acid (FFA) production in the circulation and delivery to the liver, which increases liver synthesis and secretion of TG (38). Exposure of the endothelium to high FFA levels may impair the activation of the basal and insulin-stimulated phosphatidylinositol 3-kinase (PI3K)/protein kinase B (PKB)/endothelial nitric oxide synthase signaling pathway, thereby reducing the production and activity of nitric oxide (39, 40). The inhibition of the PI3K/PKB signaling pathway may amplify the effect of the insulin-stimulated mitogen activated protein kinase (MAPK) signaling pathway with pro-atherogenic property (37), which can stimulate the production of endothelin-1 (41) and plasminogen activator inhibitor 1, control the growth and migration of vascular smooth muscle cells, and promote the aggregation of platelets (37, 42, 43). Long-term and continuous absorption of a large amount of glucose from peritoneal dialysate makes IR and compensatory hyperinsulinemia more serious in PD patients with diabetes. Therefore, IR may be the root cause of the diabetes-related difference in the impact of apo B/apo A1 ratio on mortality.

There are several limitations of this study that cannot be ignored. First, as described in the study conducted by Little et al., the lipid profile of PD patients changed dynamically and its atherogenic property progressively worsened over time (13), only using the baseline levels instead of dynamic changes for analysis in this study may underestimate the atherogenic profile over time. Second, some confounding factors that may have an impact on the prognosis, including solute transfer rate and glucose prescription, were not available in our data set, which may partially bias the results. Lastly, all participants came from a single center in China, which may limit the expansion of the conclusions to other people of different ethnicities.

In summary, we concluded that elevated apo B/apo A1 ratio level was significantly associated with increased risk of CV, all-cause, and atherosclerotic CV mortality in patients undergoing PD. Moreover, the association was especially statistically significant in patients with diabetes. In the future, more multiple-center studies are needed to verify our conclusions and evaluate whether the reduction of apo B/apo A1 ratio levels can improve the survival of PD patients, especially those with diabetes.

## Data Availability Statement

The raw data supporting the conclusions of this article will be made available by the authors, without undue reservation.

## Ethics Statement

The studies involving human participants were reviewed and approved by the Clinical Research Ethics Committee of the First Affiliated Hospital of Sun Yat-sen University. The patients/participants provided their written informed consent to participate in this study.

## Author Contributions

JY, XX, and F-XH did the concept and design the study. JY, N-YH, and Y-GQ collected the data. JY, XX, and Y-GQ analyzed the data. JY, XX, N-YH, and WC performed statistical analyses. JY drafted the manuscript. XX, N-YH, XY, H-PM, WC, and F-XH revised and completed the manuscript. All authors critically revised the manuscript, approved the final version to be published, and agreed to be accountable for all aspects of the work.

## Conflict of Interest

The authors declare that the research was conducted in the absence of any commercial or financial relationships that could be construed as a potential conflict of interest.

## Publisher’s Note

All claims expressed in this article are solely those of the authors and do not necessarily represent those of their affiliated organizations, or those of the publisher, the editors and the reviewers. Any product that may be evaluated in this article, or claim that may be made by its manufacturer, is not guaranteed or endorsed by the publisher.

## Supplementary Material

Supplementary Figure 1 Subgroup analyses of relationship of apolipoprotein B/apolipoprotein A1 ratio with the risk of CV **(A)**, all-cause **(B)**, and atherosclerotic CV **(C)** mortality stratified by several groups of clinical parameters. CI, confidence interval; CV, cardiovascular.

## Abbreviations

Apo A1: apolipoprotein A1
Apo B: apolipoprotein B
BMI: body mass index
CI: confidence interval
CIF: cumulative incidence function
CKD: chronic kidney disease
CV: cardiovascular
DBP: diastolic blood pressure
eGFR: estimated glomerular filtration rate
ESRD: end-stage renal disease
FFA: free fatty acid
HD: hemodialysis
HDL-C: high-density lipoprotein cholesterol
HR: hazard ratio
Hs-CRP: hypersensitive C-reactive protein
IR: insulin resistance
LDL-C: low-density lipoprotein cholesterol
MAPK: mitogen activated protein kinase
PD: peritoneal dialysis
PI3K: phosphatidylinositol 3-kinase
PKB: protein kinase B
Q1 to Q4: lowest to highest quartile
SBP: systolic blood pressure
SD: standard deviation
SHARP: Study of Heart and Renal Protection
SHR: subdistribution hazard ratio
SYSU: Sun Yat-sen University
TC: total cholesterol
TG: triglycerides.
