# Supplementary material for: Association of Ratio of Apolipoprotein B to Apolipoprotein A1 With Survival in Peritoneal Dialysis
Source: Front Nutr. 2022 Mar 25;9:801979. doi: 10.3389/fnut.2022.801979 (PMC8993134; doi:10.3389/fnut.2022.801979)
Supplement: Supplementary file 1 [file Data_Sheet_1.docx]

**Supplementary Material**

**Table 1** Causes of death of the study cohort

| **Causes of death** | ***n* (%)** |
| --- | --- |
| CV | 249 (49.0) |
| Atherosclerotic CV mortality | 149 (59.8) |
| Non-atherosclerotic CV mortality | 100 (40.2) |
| Other causes | 259 (51.0) |
| Infection | 105 (20.7) |
| Malignant tumor | 14 (2.8) |
| Gastrointestinal hemorrhage | 12 (2.4) |
| Failure/dyscrasia | 26 (5.1) |
| Giving up treatment | 12 (2.4) |
| Other causes | 36 (7.1) |
| Unknown causes | 54 (10.6) |

*CV, cardiovascular.*

**Table 2** Associations of apo B, apo A1, LDL-C/HDL-C ratio, and TC/HDL-C ratio with CV and all-cause mortality

|  | **HR (95% CI)**  **per 1-SD increase** |  | **Q2** |  | **Q3** |  | **Q4** |  |
| --- | --- | --- | --- | --- | --- | --- | --- | --- |
|  |  | ***P* value** | **HR (95% CI)** | ***P* value** | **HR (95% CI)** | ***P* value** | **HR (95% CI)** | ***P* value** |
| **Apo B** | | | | | | | | |
| **CV mortality** | | | | | | | | |
| Unadjusted | 1.32 (1.18–1.48) | < 0.001 | 1.05 (0.70–1.57) | 0.816 | 1.57 (1.08–2.29) | 0.019 | 1.97 (1.37–2.83) | < 0.001 |
| Final model^a^ | 1.39 (1.21–1.60) | < 0.001 | 0.88 (0.54–1.44) | 0.607 | 1.44 (0.91–2.27) | 0.120 | 2.00 (1.27–3.14) | 0.003 |
| **All-cause mortality** | | | | | | | | |
| Unadjusted | 1.23 (1.14–1.34) | < 0.001 | 0.99 (0.76–1.29) | 0.915 | 1.32 (1.02–1.71) | 0.035 | 1.61 (1.26–2.05) | < 0.001 |
| Final model^a^ | 1.21 (1.08–1.35) | 0.001 | 0.89 (0.64–1.24) | 0.491 | 1.13 (0.82–1.55) | 0.454 | 1.35 (0.98–1.85) | 0.068 |
| **Apo A1** | | | | | | | | |
| **CV mortality** | | | | | | | | |
| Unadjusted | 0.43 (0.84–1.08) | 0.952 | 1.02 (0.72–1.46) | 0.900 | 1.00 (0.70–1.44) | 0.991 | 0.99 (0.70–1.39) | 0.934 |
| Final model^a^ | 1.08 (0.93–1.26) | 0.324 | 1.07 (0.69–1.67) | 0.750 | 1.23 (0.79–1.91) | 0.372 | 1.41 (0.91–2.17) | 0.124 |
| **All-cause mortality** | | | | | | | | |
| Unadjusted | 0.87 (0.79–0.95) | 0.001 | 0.87 (0.68–1.10) | 0.241 | 0.87 (0.68–1.11) | 0.248 | 0.74 (0.58–0.95) | 0.016 |
| Final model^a^ | 0.61 (0.87–1.09) | 0.972 | 0.87 (0.64–1.19) | 0.381 | 1.05 (0.77–1.42) | 0.776 | 0.98 (0.72–1.33) | 0.911 |
| **LDL-C/HDL-C** | | | | | | | | |
| **CV mortality** | | | | | | | | |
| Unadjusted | 1.25 (1.13–1.39) | < 0.001 | 0.99 (0.68–1.44) | 0.944 | 1.37 (0.95–1.97) | 0.089 | 1.64 (1.15–2.33) | 0.006 |
| Final model^a^ | 1.29 (1.13–1.48) | < 0.001 | 1.55 (0.98–2.45) | 0.059 | 1.60 (1.03–2.49) | 0.038 | 1.91 (1.23–2.96) | 0.004 |
| **All-cause mortality** | | | | | | | | |
| Unadjusted | 1.22 (1.13–1.31) | < 0.001 | 0.91 (0.70–1.18) | 0.484 | 1.19 (0.93–1.53) | 0.171 | 1.49 (1.17–1.89) | 0.001 |
| Final model^a^ | 1.19 (1.07–1.32) | 0.001 | 1.28 (0.93–1.76) | 0.125 | 1.26 (0.93–1.72) | 0.141 | 1.48 (1.09–2.01) | 0.011 |
| **TC/HDL-C** | | | | | | | | |
| **CV mortality** | | | | | | | | |
| Unadjusted | 1.24 (1.12–1.37) | < 0.001 | 1.07 (0.74–1.57) | 0.713 | 1.45 (1.01–2.10) | 0.045 | 1.75 (1.23–2.50) | 0.002 |
| Final model^a^ | 1.22 (1.06–1.40) | 0.004 | 1.23 (0.78–1.94) | 0.369 | 1.81 (1.16–2.81) | 0.009 | 1.54 (0.98–2.40) | 0.061 |
| **All-cause mortality** | | | | | | | | |
| Unadjusted | 1.27 (1.18–1.36) | < 0.001 | 0.98 (0.75–1.27) | 0.870 | 1.23 (0.95–1.59) | 0.118 | 1.78 (1.40–2.26) | < 0.001 |
| Final model^a^ | 1.19 (1.08–1.32) | 0.001 | 1.02 (0.74–1.40) | 0.911 | 1.38 (1.01–1.89) | 0.046 | 1.40 (1.03–1.90) | 0.031 |

*We indicated the lowest quartile (Q1) as the reference group.*

*Apo A1, apolipoprotein A1; Apo B, apolipoprotein B; CI, confidence interval; CV, cardiovascular; HDL-C, high-density lipoprotein cholesterol; HR, hazard ratio; LDL-C, low-density lipoprotein cholesterol; Q1 to Q4, lowest to highest quartile; SD, standard deviation; TC, total cholesterol.*

*^a^Adjusted for age, sex, diabetes, a history of cardiovascular events, body mass index, systolic blood pressure, hemoglobin, serum albumin, hypersensitive C-reactive protein, estimated glomerular filtration rate, total Kt/V, and statin use.*

**Table 3** Associations of apo B, apo A1, LDL-C/HDL-C ratio, and TC/HDL-C ratio with atherosclerotic and non-atherosclerotic CV mortality

|  | **SHR (95% CI)**  **per 1-SD increase** |  | **Q2** |  | **Q3** |  | **Q4** |  |
| --- | --- | --- | --- | --- | --- | --- | --- | --- |
|  |  | ***P* value** | **SHR (95% CI)** | ***P* value** | **SHR (95% CI)** | ***P* value** | **SHR (95% CI)** | ***P* value** |
| **Apo B** | | | | | | | | |
| **Atherosclerotic CV mortality** | | | | | | | | |
| Unadjusted | 1.55 (1.36–1.77) | < 0.001 | 1.18 (0.67–2.08) | 0.563 | 2.15 (1.28–3.60) | 0.004 | 2.76 (1.68–4.54) | < 0.001 |
| Final model^a^ | 1.59 (1.34–1.88) | < 0.001 | 0.97 (0.47–2.01) | 0.940 | 1.84 (0.94–3.58) | 0.073 | 2.63 (1.37–5.03) | 0.004 |
| **Non-atherosclerotic CV mortality** | | | | | | | | |
| Unadjusted | 0.94 (0.77–1.13) | 0.501 | 0.92 (0.52–1.62) | 0.768 | 0.99 (0.56–1.74) | 0.963 | 1.12 (0.65–1.92) | 0.694 |
| Final model^a^ | 0.98 (0.79–1.20) | 0.813 | 0.89 (0.41–1.94) | 0.771 | 0.96 (0.46–2.00) | 0.918 | 1.20 (0.60–2.40) | 0.613 |
| **Apo A1** | | | | | | | | |
| **Atherosclerotic CV mortality** | | | | | | | | |
| Unadjusted | 0.85 (0.72–1.00) | 0.053 | 1.13 (0.74–1.75) | 0.571 | 0.94 (0.60–1.48) | 0.785 | 0.72 (0.45–1.15) | 0.168 |
| Final model^a^ | 1.07 (0.87–1.31) | 0.524 | 1.21 (0.69–2.12) | 0.506 | 1.43 (0.82–2.51) | 0.211 | 1.25 (0.69–2.26) | 0.466 |
| **Non-atherosclerotic CV mortality** | | | | | | | | |
| Unadjusted | 1.12 (0.94–1.34) | 0.208 | 0.82 (0.44–1.52) | 0.523 | 1.08 (0.60–1.94) | 0.798 | 1.46 (0.86–2.47) | 0.157 |
| Final model^a^ | 1.10 (0.86–1.41) | 0.425 | 0.81 (0.38–1.76) | 0.601 | 0.94 (0.44–2.04) | 0.886 | 1.55 (0.75–3.21) | 0.234 |
| **LDL-C/HDL-C** | | | | | | | | |
| **Atherosclerotic CV mortality** | | | | | | | | |
| Unadjusted | 1.43 (1.27–1.61) | < 0.001 | 1.00 (0.58–1.74) | 0.991 | 1.80 (1.10–2.96) | 0.020 | 2.57 (1.60–4.10) | < 0.001 |
| Final model^a^ | 1.46 (1.26–1.69) | < 0.001 | 1.35 (0.68–2.71) | 0.393 | 1.84 (0.98–3.45) | 0.059 | 2.48 (1.34–4.58) | 0.004 |
| **Non-atherosclerotic CV mortality** | | | | | | | | |
| Unadjusted | 0.84 (0.67–1.05) | 0.132 | 0.98 (0.58–1.65) | 0.940 | 0.92 (0.54–1.60) | 0.779 | 0.72 (0.40–1.29) | 0.270 |
| Final model^a^ | 0.90 (0.72–1.13) | 0.378 | 1.62 (0.80–3.28) | 0.180 | 1.09 (0.52–2.31) | 0.813 | 0.98 (0.48–1.98) | 0.951 |
| **TC/HDL-C** | | | | | | | | |
| **Atherosclerotic CV mortality** | | | | | | | | |
| Unadjusted | 1.40 (1.23–1.61) | < 0.001 | 1.34 (0.77–2.34) | 0.305 | 2.26 (1.35–3.81) | 0.002 | 3.14 (1.91–5.18) | < 0.001 |
| Final model^a^ | 1.36 (1.17–1.58) | < 0.001 | 1.36 (0.67–2.73) | 0.391 | 2.45 (1.29–4.66) | 0.006 | 2.26 (1.18–4.32) | 0.014 |
| **Non-atherosclerotic CV mortality** | | | | | | | | |
| Unadjusted | 0.81 (0.65–1.02) | 0.072 | 0.89 (0.53–1.48) | 0.649 | 0.82 (0.48–1.43) | 0.490 | 0.69 (0.39–1.23) | 0.207 |
| Final model^a^ | 0.83 (0.66–1.06) | 0.143 | 1.08 (0.55–2.16) | 0.816 | 0.92 (0.45–1.89) | 0.823 | 0.73 (0.36–1.47) | 0.371 |

*We indicated the lowest quartile (Q1) as the reference group.*

*Apo A1, apolipoprotein A1; Apo B, apolipoprotein B; CI, confidence interval; CV, cardiovascular; HDL-C, high-density lipoprotein cholesterol; LDL-C, low-density lipoprotein cholesterol; Q1 to Q4, lowest to highest quartile; SD, standard deviation; SHR, subdistribution hazard ratio; TC, total cholesterol.*

*^a^Adjusted for age, sex, diabetes, a history of cardiovascular events, body mass index, systolic blood pressure, hemoglobin, serum albumin, hypersensitive C-reactive protein, estimated glomerular filtration rate, total Kt/V, and statin use.*
