# Supplementary figures and images for: Association of Ratio of Apolipoprotein B to Apolipoprotein A1 With Survival in Peritoneal Dialysis
Source: Front Nutr. 2022 Mar 25;9:801979. doi: 10.3389/fnut.2022.801979 (PMC8993134; doi:10.3389/fnut.2022.801979)

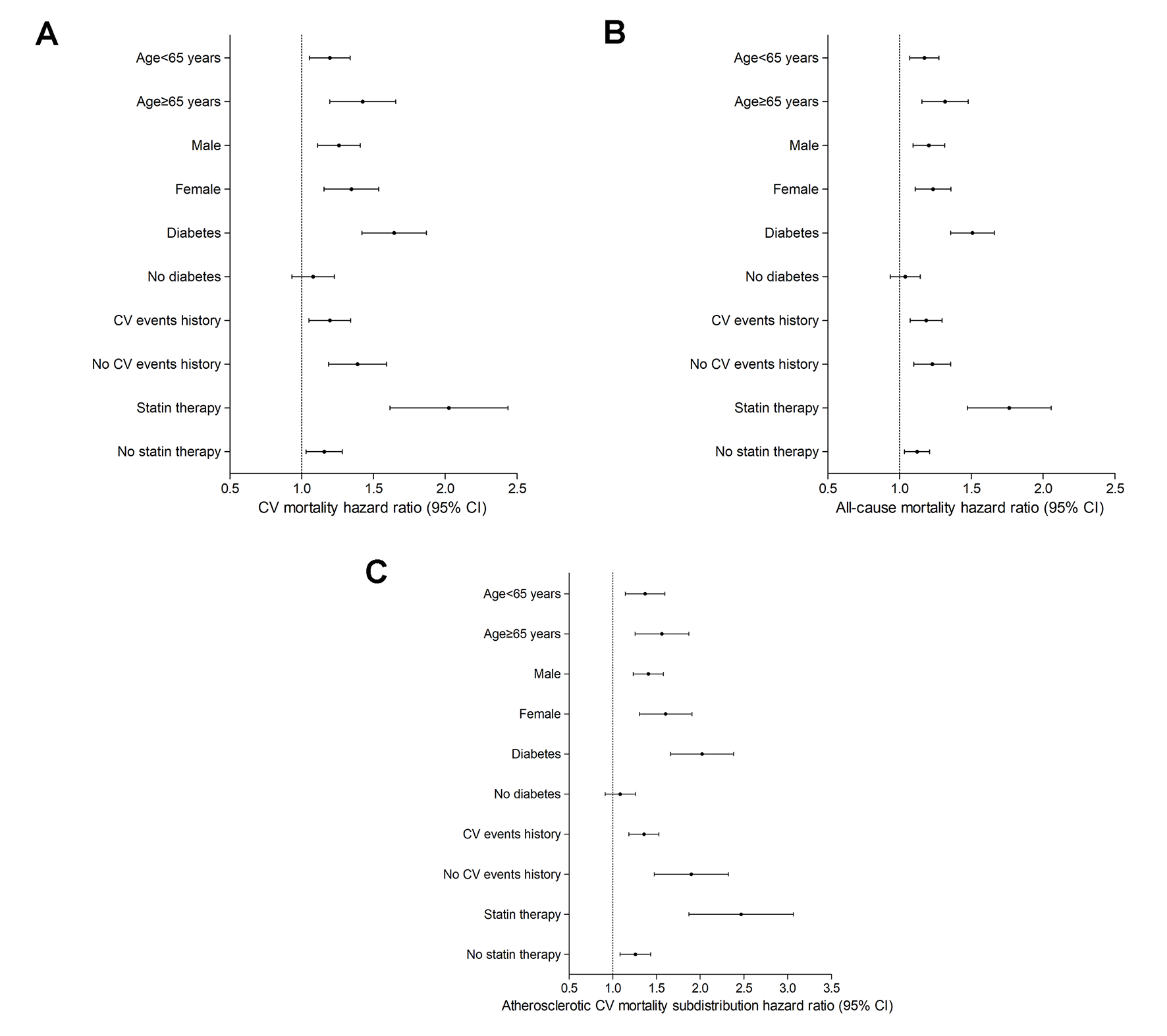

Supplement: Supplementary Figure 1 — Subgroup analyses of relationship of apolipoprotein B/apolipoprotein A1 ratio with the risk of CV (A), all-cause (B), and atherosclerotic CV (C) mortality stratified by several groups of clinical parameters. CI, confidence interval; CV, cardiovascular. [file Image_1.TIF]
